# Ethnic disparity in Israel impacts long-term results after heart transplantation

**DOI:** 10.1186/s13584-018-0271-7

**Published:** 2019-01-14

**Authors:** Yael Peled, Ron Loewenthal, Yigal Kassif, Eugenia Raichlin, Arwa Younis, Anan Younis, Eyal Nachum, Dov Freimark, Jacob Lavee

**Affiliations:** 1 The Olga and Lev Leviev Heart Center, Sheba Medical Center Tel Hashomer, Heart Transplantation Unit, Heart Failure Institute, 52621 Ramat Gan, Israel; 2 The Sackler School of Medicine, Tel Aviv University, Tel Aviv, Israel; 3 Tissue Typing Laboratory Sheba Medical Center, Ramat Gan, Israel; 4 Cardiology Department, Loyola University Medical Center, Maywood, IL USA

**Keywords:** Heart transplantation, Ethnicity, Jews, Arabs, Cardiac allograft vasculopathy, Cardiovascular mortality

## Abstract

**Background:**

Ethnicity may affect graft longevity and recipient mortality after heart transplantation (HTx). We hypothesized that differences in ethnic origin between Arabs and Jews undergoing HTx in Israel may contribute to differences in long-term outcomes.

**Methods:**

The study population comprised all 254 patients who underwent HTx between 1991 and 2017 in a tertiary medical center located in the center of Israel. Patients were categorized as either Jews (226 patients, 89%) or Arabs (28 patients, 11%). The primary end point was cardiac allograft vasculopathy (CAV), secondary end points were cardiovascular (CV) mortality and the combined end point of CAV/CV mortality.

**Results:**

In comparison with Jews, Arab patients were significantly younger (ave. age 42 vs. 50) and had shorter in-hospital stay (45 vs. 80 days). However, Kaplan-Meier survival analysis showed that at 10 years of follow-up CAV rates were significantly higher among Arabs (58%) compared with Jews (23%; log-rank *P* = 0.01) for the overall difference during follow-up. Similar results were shown for the separate end point of CV mortality and the combined end point of CAV/CV mortality. Multivariate analysis, which controlled for age, gender, statin treatment, and other potential confounders, showed that Arab recipient ethnic origin was associated with a significant > 2.5-fold (*p* = 0.01) increase in the risk for CAV; a > 4-fold increase in the risk for CV mortality (*p* = 0.001); and approximately 4-fold increase in the risk for the combined end point (p = 0.001). These findings were validated by propensity score analysis.

**Conclusions:**

Our data suggest that Arab ethnic origin is associated with a significantly increased risk for CAV and mortality following HTx. Suggested explanations contributing to ethnic disparities in Israel include socioeconomic, environmental and genetic factors. Further studies are required to evaluate whether more aggressive risk factor management in the Israeli Arab population following HTx would reduce CAV and CV mortality in this high-risk population. Increased awareness and early intervention of the Israeli healthcare system and cooperation with the Arab community is of paramount importance.

## Background

Heart transplantation (HTx) is the gold standard curative therapy for selected patients with end-stage heart failure. Since the first HTx in 1967 [1], survival and outcomes have improved considerably. Multiple factors have been identified that contribute to improved outcomes [2]. Several studies have reported ethnicity as a predictor of graft longevity and recipient mortality in HTx [3–5]. Available evidence suggests that the survival of African American patients after HTx is lower compared with other ethnic groups [6]. Furthermore, long-term survival after HTx has improved across eras in Caucasian recipients but not in their African American or Hispanic-Latino counterparts, suggesting that racial/ethnic disparities in long-term survival after HTx have worsened over time [4]. Contributing factors are socioeconomic, immunologic, and pharmacogenetic [7].

Israeli Arabs and Jews are both heterogeneous groups that together comprise the vast majority of Israel’s population (Jews 74.8% and Arabs 20.7%) [8]. Both Ashkenazi and Sephardic Jews have been knowingly segregated for generations, and as such have diseases typically attributed almost solely to these separate populations (Tay-Sachs, Canavan) [9, 10]. Israeli Arabs are also known for their segregation and consanguinity and accordingly show an increase prevalence of certain diseases (Thalasemia major, Krabbe) [11, 12].

The rate of HTx and organ donations among Israeli Arabs and Jews are proportional to their representation in the general population. We hypothesized that Israeli Arabs and Jews undergoing HTx may have different characteristics because of differences in their environmental and genetic background. Therefore, we aimed to evaluate these differences and their impact on HTx outcomes.

## Materials and methods

### Study population and registry design

Between 1991 and 2017, 285 patients who underwent HTx were enrolled and prospectively followed-up in the tertiary center Sheba HTx Registry. Excluded from further analysis were children (*n* = 11), patients who underwent HTx in China and whose donors might have been executed prisoners (*n* = 13), in accordance with current ethical guidelines of transplantation societies [13], and additional patients whose ethnic origin was unknown (*n* = 7). Thus, the present study population comprised 254 patients.

### Definitions and endpoints

#### Ethnic groups

Ethnic groups were categorized according to the Statistical Abstract of Israel [9]: Jews; Arab Moslems (including Circassians), Arab Christians (including Armenians), and Druze; others: non-Arab Christians, members of other religions, and those not classified by religion in the Population Registry.

#### Immunosuppression

All patients were treated with a triple-drug regimen. Maintenance immunosuppression comprised combination therapy including prednisone, an antimetabolite, and a calcineurin inhibitor. Conversion to everolimus was based on the patient’s risk profile, including cytomegalovirus infection, renal failure, allograft vasculopathy, and malignancy risk. All patients also received induction therapy consisting of anti-thymocyte globulin.

#### Rejections, surveillance and classification

Routine endomyocardial biopsies (EMBs) were performed every week for the first 4 weeks post HTx, twice a month during the second and third months, once a month for the following 3 months, and thereafter every 3 months until the end of the first year. From the end of the first year until the end of the fifth year, biopsies were carried out annually. Rejections were diagnosed by routine or clinically indicated EMB and were classified according to the revised International Society of Heart and Lung Transplantation (ISHLT) classification system for rejection [14]. For each patient, we calculated a total rejection score (TRS) normalized by dividing the number of EMBs with signs of rejection by the total number of EMBs taken during the study period. Also, all patients who had any rejection on EMB were considered to have any rejection score (ARS) of 1; and we normalized that for each patient by dividing by the total number of EMBs taken during the study period [15].

#### Cardiac allograft vasculopathy

The institutional post-transplant care protocol includes annual invasive coronary angiography for the first five years following HTx along with echocardiogram and right heart catheterization. Cardiac allograft vasculopathy (CAV) was diagnosed by coronary angiography and invasive hemodynamic assessment was performed annually, along with clinical assessment and echocardiography, combined according to the recommended nomenclature for CAV of the ISHLT consensus statement [16].

#### Outcome measures

The primary outcome measure of this study was the development of CAV; secondary outcome measures included cardiovascular (CV) mortality and the combined end point of CAV or long-term CV mortality (mean follow-up of 9.2 ± 4.2 years). Cardiovascular mortality included death due to: CAV, acute rejection, graft failure, cerebrovascular and sudden cardiac death.

##### Statistical analysis

Descriptive statistics were produced using means and standard deviations for continuous variables (e.g. age), and frequencies for categorical variables (e.g. ethnic origin). To examine differences between groups in continuous variables, Mann-Whitney procedures were conducted to avoid bias for non-normal distributions. To examine differences between groups in categorical variables, Chi-Square tests were conducted.

The Kaplan–Meier estimator was used to assess the time to the first occurrence of each endpoint by the recipient ethnic origin, and groups were compared using the log-rank test. Multivariable Cox proportional hazard regression analysis was used to evaluate the association between the recipient ethnic origin and the first occurrence of endpoints during follow-up. Covariates included in the multivariate models were identified using the best subset procedure among variables that were predictive of the endpoint and were unbalanced among the two groups (candidate covariates are listed in Tables 1 and 2). Overall, due to significant differences between groups, the following variables were statistically controlled in further analyses: gender, age and statin treatment of the recipients.

**Table 1:** Characteristics of patients by ethnicity

	Ethnic origin of recipient		*P*-value
	Jews (*N =* 226)	Arabs (*N =* 28)	
Gender - recipient [male, %]	84	64	0.01
Gender - donor [male, %]	70	73	0.90
Age - recipient (years)	50 ± 12	42 ± 14	0.001
Age - donor (years)	33 ± 12	35 ± 12	0.46
Weight - recipient (kg)	73 ± 14	74 ± 16	0.61
Weight - donor (kg)	75 ± 18	77 ± 21	0.52
Height - recipient (cm)	171 ± 11	172 ± 9	0.78
Height - donor (cm)	174 ± 13	176 ± 7	0.50
BMI - recipient	25 ± 4	25 ± 5	0.51
BMI - donor	25 ± 5	26 ± 6	0.50
Etiology of HTx (IHD, %)	61	43	0.07
Hypertension (%)	37	36	0.90
Diabetes (%)	19	28	0.22
Dyslipidemia (%)	48	36	0.21
Past Smoker (%)	43	43	0.98
Listing status (%)			
Status 1	69	80	0.27
Status 2	31	20	
Creatinine (mg/dL)	1.6 ± 3.7	1.2 ± 0.5	0.55
Bilirubin (mg/dL)	1.3 ± 2.4	1.2 ± 0.7	0.83
Systolic pulmonary artery pressure (mmHg)	51 ± 19	48 ± 18	0.45
Diastolic pulmonary artery pressure (mmHg)	25 ± 11	26 ± 10	0.63
Mean pulmonary artery pressure (mmHg)	35 ± 14	36 ± 13	0.88
Pulmonary capillary wedge pressure(mmHg)	25 ± 11	25 ± 9	0.90
Cardiac output (L/min)	3.6 ± 1.0	3.4 ± 1.5	0.43
Pulmonary vascular resistance (Wood)	3.2 ± 2.2	2.8 ± 2.3	0.58
ICD (%)	42	41	0.89
Family history of IHD (%)	52	41	0.26
LVAD bridge to HTx (%)	16	18	0.78
PRA > 30%	0.5	0.0	0.97
CMV mismatch	64	56	0.48
Blood type			
A (%)	45	40	0.90
AB (%)	12	12	
B (%)	17	16	
O (%)	25	32	

Continuous variables and categorical variables are presented as mean ± standard deviation and percentage, respectively

*BMI* body mass index, *HTx* heart transplantation, *CMV* cytomegalovirus, *ICD* implantable cardioverter defibrillator, *IHD* ischemic heart disease, *LVAD* left ventricular assist device, *PRA* panel of reactive antibodies

**Table 2:** Patients’ Operative and Post-Operative Data by Ethnic Origin

	Ethnic origin of recipient		*P*-value
	Jews (*N =* 226)	Arabs (*N =* 28)	
Operative Data			
Ischemic time (minutes)	160 ± 45	153 ± 35	0.85
Primary graft dysfunction	33	36	0.80
Time from admission to discharge(days)	80 ± 120	43 ± 45	0.007
Time from HTx to discharge (days)	28 ± 46	18 ± 9	0.28
Early complications ^a^	58	55	0.74
In-hospital death	13	18	0.51
Post-Operative Data			
Statin after HTx	91	68	< 0.001
Baseline LDL after HTx^b^	109 ± 34	132 ± 38	< 0.01
Hypertension after HTx	65	53	0.44
Diabetes after HTx	32	37	0.68
CMV disease	22	9	0.14
Immunosuppression			0.55
Cyclosporine- based	59	50	
Tacrolimus-based	39	50	
Everolimus-based	2	0	

Continuous variables and categorical variables are presented as mean ± standard deviation and percentage, respectively

*HTx* heart transplantation, *LDL* low density lipoprotein, *CMV* cytomegalovirus

Early complications ^a^ – prolonged ventilation, sepsis, severe coagulopathy, cerebrovascular accident, prolonged chest tubes, early wound infection. LDL^b^ - as measured at 3 months following HTx

To further validate our findings, we calculated a propensity score for the probability of ethnic origin (Jew/Arab) using binary logistic regression. We included the covariates that were found to be significantly different by ethnic origin according to Tables 1 to 3. Data were analyzed with SPSS software version 23. A two-sided 0.05 significance level was used for hypothesis testing.

**Table 3:** Predicting CAV, CV mortality and combined end point of CAV/CV mortality by ethnic origin: multivariate cox proportional hazard model, and propensity score analysis

	CAV				CV mortality				CAV/CV mortality			
	Multivariate (Cox)		Propensity		Multivariate (Cox)		Propensity		Multivariate (Cox)		Propensity	
	HR	p	HR	P	HR	p	HR	p	HR	p	HR	p
Ethnic (Arabs)	.692 [1.47,3.91]	0.01	2.43 [1.63,3.23]	0.016	4.78 [3.55,6.01]	0.001	1.47 [1.22,1.92]	0.001	3.81 [2.12,5.11]	0.001	3.75 [2.02,5.24]	0.001
Age	.980 [0.76,1.21]	0.16			0.99 [0.78,1.22]	0.86			1.01 [0.38,1.62]	0.64		
Sex (Male)	1.69 [0.89,2.49]	0.28			5.25 [3.98,6.51]	0.048			0.85 [0.95,4.56]	0.74		
LDL	1.29 [0.49,2.09]	0.72			1.03 [1.01,1.05]	0.037			1.01 [0.59,1.43]	0.49		

*CAV*, Cardiac allograft vasculopathy, *CV*, Cardiovascular *LDL*, Low-density lipoprotein

## Results

### Clinical characteristics of study patients by ethnic origin

The present study population comprised 254 patients between the age of 17 and 70 years (45 ± 13), of whom 82% (*n* = 208) were male.

Among the study population 89% were Jews (*n* = 226) and 11% were Arabs (*n* = 28). The baseline characteristics of recipients by ethnic origin are summarized in Table 1. Arabs recipients were significantly younger than Jewish recipients (42 ± 14 vs. 50 ± 12, respectively, *p* = 0.01). The mean age of the donors did not differ between the ethnic groups. Donor ethnicity data were available only for 166 HTx patients of the total cohort. Of the 139 Jewish recipients, 109 had Jewish and 30 had Arab donors. Of the 27 Arab recipients, 25 had Jewish and 2 had Arab donors. The rate of organ donations among Israeli Arabs and Jews in the current study was found to be proportional to their representation in the general population.

In both groups, the percentage of men was higher than that of women, but this difference was more pronounced among Jewish recipients than among the Arabs. Ischemic heart disease was a more common cause for HTx among Jews than among Arabs. No differences were found between the two groups relating to donors’ characteristics. Pre-transplant donor-specific antibodies (DSAs) were negative for all patients. Crossmatch in our series was negative for all patients.

Operative and post-operative data are summarized in Table 2*.* Length of hospitalization was longer among Jews compared to Arabs recipients (80 ± 120 vs. 43 ± 45 days, respectively; *p* = 0.007), due to longer pre-HTx waiting time. In addition, rates of statin therapy after HTx were higher among Jews compared to Arabs (91% vs. 68%, respectively; *p* < 0.001). Consistently, LDL levels following HTx were higher among Arabs. Immunosuppression protocol did not differ between the ethnic groups.

### Predictors of heart transplantation outcomes

#### Cav

**Fig. 1:**
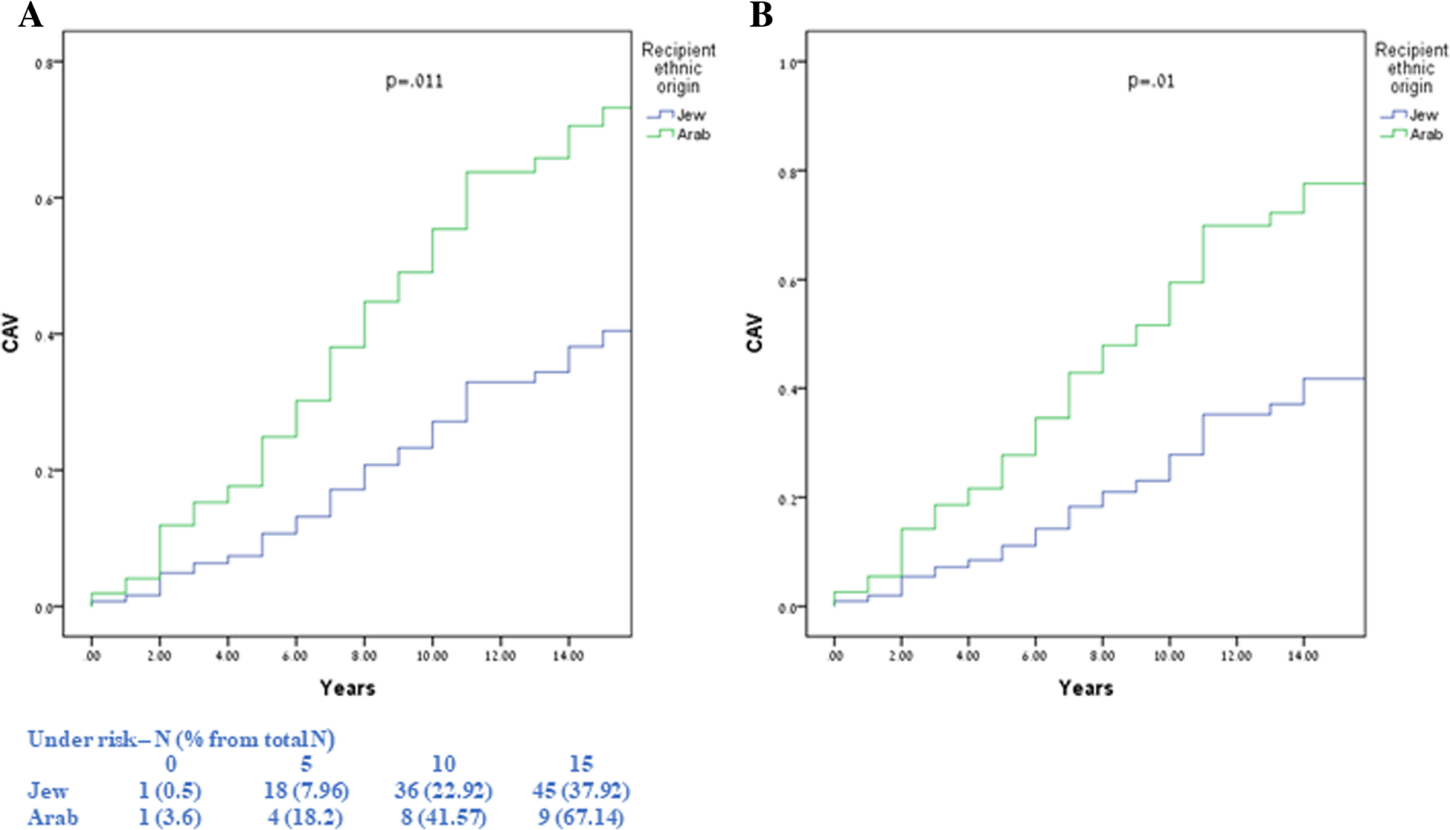
CAV survival according to recipient ethnic origin using Kaplan-Meier analysis (**a**) and propensity score modeling (**b**)

Kaplan-Meier survival analysis showed that the rate of CAV was significantly higher among Arab compared with Jewish recipients. At 10 years of follow-up the rate of CAV was 58% among Arabs compared to 24% among Jews (log-rank *p* < 0.011) for the overall differences during follow-up (Fig. 1a).

In a multivariate model predicting for CAV by ethnic origin and covariates, CAV rates were independently higher among Arab recipients compared with Jews, both by multivariate (HR = 2.69, 95% CI [1.47, 3.91], *p* = 0.01) and controlled propensity score (HR = 2.43, 95% CI [1.63, 3.23], *p* = 0.016), as shown in Table 3 and in Fig. 1b.

#### Risk of CV mortality by ethnic origin

**Fig. 2:**
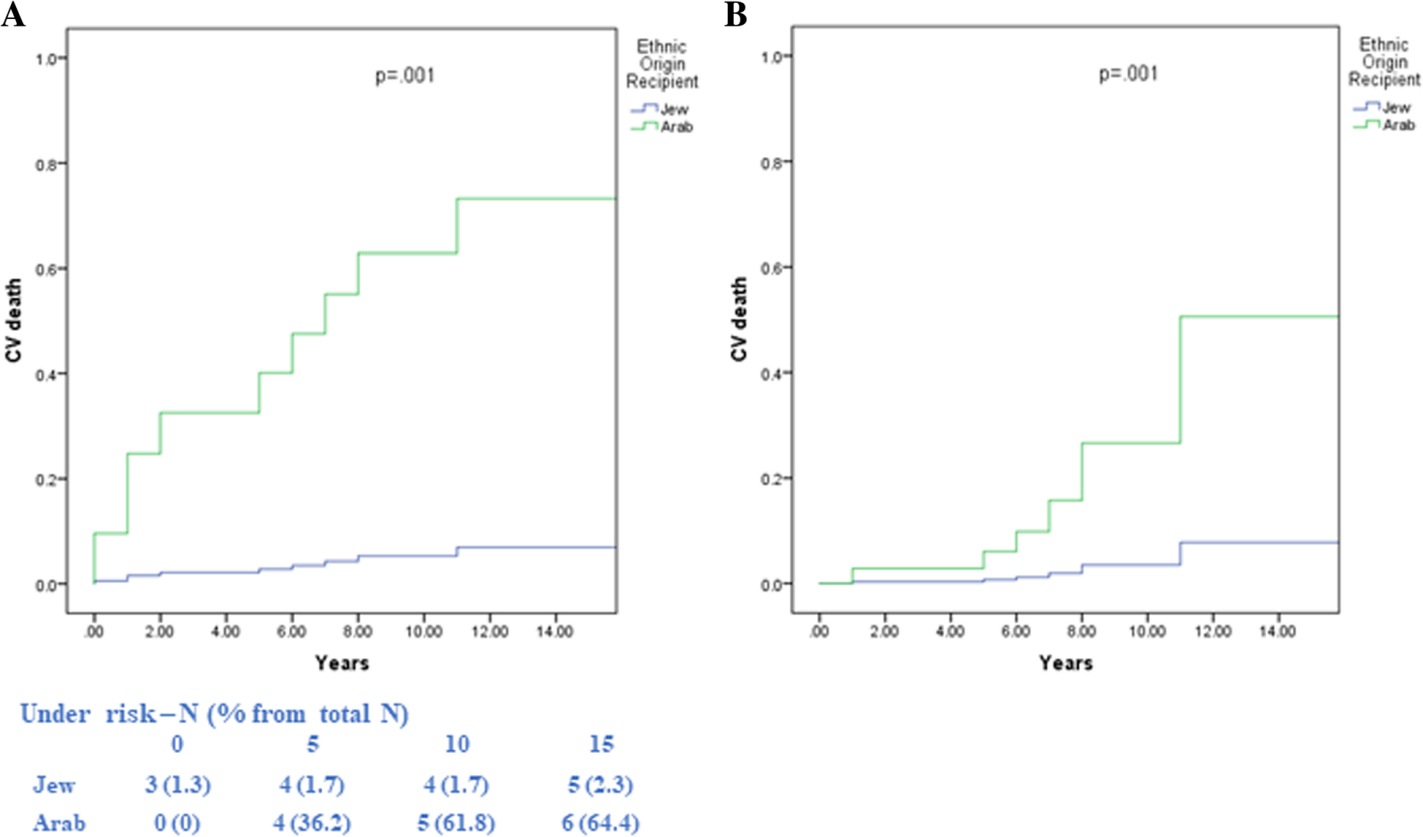
Cardiovascular survival according to recipient ethnic using Kaplan-Meier analysis (**a**) and propensity score modeling (**b**)

Kaplan-Meier survival analysis showed that the rate of CV mortality among Arab recipients was higher than among the Jewish recipients. The corresponding rates of CV mortality at 10 years of follow-up were 65% among the Arabs recipients vs. 5.9% among the Jewish recipients (log-rank *P* = 0.001; Fig. 2a).

In a multivariate model predicting CV mortality by ethnic origin and covariates, and controlling for age, gender, and a wide range of potential confounder, CV death rates were higher in the Arab recipients compared with the Jewish recipients, both by multivariate analysis (HR = 4.78, 95% CI [3.55, 6.01], *p* = 0.001) and controlled propensity score (HR = 1.47, 95% CI [1.22, 1.92], p = 0.001), as shown in Table 3 and Fig. 2b.

#### Risk of combined end point of CAV or CV mortality by ethnic origin

**Fig. 3:**
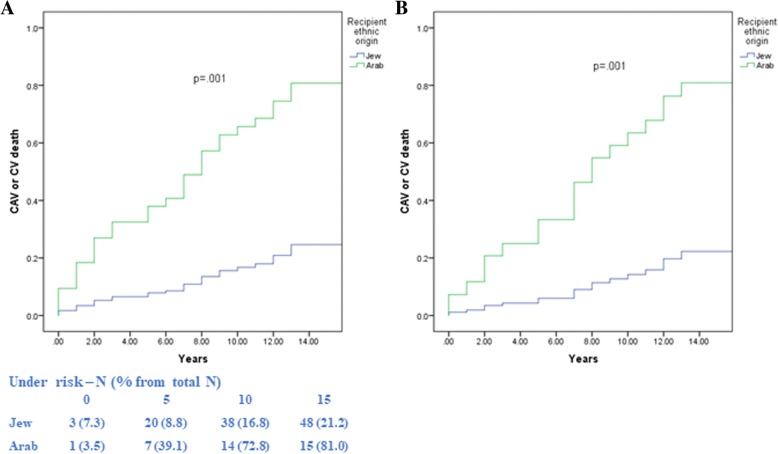
Kaplan Meier survival curve of the combined end point of CAV or CV mortality according to recipient ethnic origin using Kaplan-Meier analysis (**a**) and propensity score modeling (**b**)

Kaplan-Meier survival analysis showed that the combined endpoint of CAV or CV mortality was significantly higher in Arab patients. The corresponding rates of combined end point of CAV or CV mortality at 10 years of follow-up were: 75% among the Arab recipients vs. 15% among the Jewish recipients (log-rank *P* < 0.001; Fig. 3).

In a multivariate model predicting survival for the combined end point of CAV or CV mortality by ethnic race and covariates, rates were higher among the Arabs compared with the Jewish recipients, both by multivariate (HR = 3.81, 95% CI [2.12, 5.11], p = 0.001) and controlled propensity score (HR = 3.75, 95% CI [2.02, 5.24], p = 0.001), as shown in Table 3 and Fig. 3b.

#### Rejections

No differences were found in the rejection rates according to the patients’ ethnic groups, nor in the time to first major rejection (TRS 0.55 ± 0.46 vs. 0.54 ± 0.27, *p* = 0.93; ARS 0.46 ± 0.35 vs. 0.47 ± 0.25, *p* = 0.96; for Jews and Arab recipients, respectively).

## Discussion

Israeli Arabs and Jews are both heterogeneous groups that together comprise the vast majority of Israel’s population. Our study provides several important novel findings regarding ethnic differences between Arabs and Jews following HTx. We have shown that: 1) Arab recipient origin is associated with significant and independent increased risk for CAV; 2) Cardiovascular mortality and the combined end point of CAV/CV mortality are also significantly higher in the Arab recipients; and 3) There were no significant differences in rejection rates between the two groups. Unique to our patients is the uniform accessibility to transplant care and eligibility to the same treatments and follow-up. It should be emphasized that these findings are in spite of similar treatment recommendations provided to both groups free of charge on the basis of the Israeli National Health Insurance policy.

### Recipient race and clinical characteristics

HTx is the treatment of choice for patients suffering from end stage heart failure despite maximum medical therapy, with survival continuing to improve over time [2]. However, reports confirm that the benefit and improved results of HTx are not uniform since certain racial/ethnic minorities have inferior outcomes [3, 4, 6, 7]. Of the HTx recipients, Israeli Arabs have a trend toward greater prevalence of non-ischemic cardiomyopathy, which seems to be a more aggressive form of cardiomyopathy. While most of the recipients are male, the proportion of females is higher, with younger age at transplantation. These findings are in line with a recent study suggesting a higher prevalence of non-ischemic cardiomyopathy characterized by early onset and rapid deterioration among Jewish and Arab patients with heart failure who have ICD/CRTD implantations [17].

### Ethnicity and mortality

Life expectancy in Israel is higher in the Jewish population than among Arabs. In 2014, average life expectancy at birth was 81.1 years for Jewish men, 84.5 years for Jewish women, 76.9 years for Arab men and 81.2 years for Arab women [8]. Mortality differentials between Arabs and Jews declined at ages below 45 years and increased among older people in whom heart disease, diabetes and cancer are the major contributors for the increasing inequality among the elderly [2, 18]. In the present study, although age at HTx was younger for the Arab recipients with a tendency toward more female recipients among this population, CV mortality was higher among the overall Arabs recipients with a > 4-fold increase in the risk for CV mortality after adjusting for covariates. No differences were found in overall mortality between these two ethnic groups.

A recent study of cystic fibrosis patients who underwent lung transplantation showed better correlated survival among the ethnic Jewish population [19]. This study also showed that bronchiolitis obliterans syndrome was more common and appeared earlier in the Arab compared with the Jewish population.

Ethnic related differences in mortality among patients following HTx have been previously reported, albeit in other ethnic groups. A more than two-decade follow-up study of > 39, 000 HTx patients found that black recipients had an increased risk of death when compared with white recipients after multivariable adjustment for recipient, transplant, and socioeconomic factors [20]. Although overall survival of HTx patients has improved during the last decade, Singh et al. showed that among 36,784 HTx recipients, long-term survival had improved in white but not in black or Hispanic recipients, resulting in a more marked disparity in outcomes in the current era [4]. The disparity in outcomes in minority populations is not unique to HTx, as ethnic minorities have been associated with reduced survival also after other solid organ transplantations [21].

### Ethnicity and CAV

CAV remains a leading cause of graft loss and mortality among late survivors of HTx. A novel finding of our study is that the ethnic origin of Arab recipients is independently associated with a significant > 2-fold increase in the risk for CAV.

It has been previously reported that African American HTx recipients have increased risk for CAV, and shorter time to the development of CAV compared with Hispanic-Latino and Caucasian recipients [6, 22]. Similarly, a study including 5211 pediatric HTx recipients included in the OPTN/UNOS Database showed that the African American race was highly associated with shorter CAV-free survival [23].

Differences in CV mortality and cerebral events have been reported in the Israel National Survey, suggesting higher rates of mortality from all heart disease in Arab men and women compared with their Jewish counterparts (15% vs. 27%, respectively) [8, 24]. While the higher risk of CAV in African Americans is likely related to the increased burden of acute cellular rejection, a known risk factor for earlier development of CAV, no differences in rejection rates related to a patient’s ethnic group were found in our study [6, 15]. To the best of our knowledge, the present study is the first to address the impact of these ethnicities on outcomes after HTx.

Because CAV is a major cause of graft loss and mortality, prevention of CAV is critical in order to improve graft survival. The present findings suggest that more aggressive attempts should be adopted. Transplant recipients with known risk factors for CAV should be monitored closely and receive early intervention to reduce CAV risk. These interventions should focus on education of patients, strengthening the professional relationship between community medicine and the tertiary center. Implementation of prevention programs to reduce risk factors, particularly diabetes and obesity. Individualizing the cardiovascular follow up protocol emphasizing on early vasculopathy assessment including frequent oriented visits and more frequent use of non-invasive modalities. Primary prevention treatment with aspirin should also be considered [25].

### Factors contributing to racial/ethnic disparities in post-transplant outcomes

Suggested explanations contributing to ethnic disparities in Israel include socioeconomic, environmental and genetic factors [6, 26, 27]. The Jewish and Arab populations differ in social, cultural, economic and genetic characteristics, as well as in health characteristics. Arabs in Israel have lower socioeconomic status and poorer health awareness [28, 29], that have previously been shown to be related to increased mortality and CV morbidity.

Dietary patterns might also play a role. Jews and Arabs from the same region in Israel exhibit major differences in food consumption. A high intake of the foods historically produced by the rural Arab population, now demonstrate modifications that have reduced the healthy properties of the traditional Arab diet (e.g. replacement of whole grains with refined grains, increased consumption of meat dishes/animal fat) [30]. These dietary differences contribute to the disparity seen in the current LDL levels between ethnic groups. Katler et al. have consistently shown that levels of HDL and triglycerides were significantly worse among Arabs compared with Jews in a study cohort of > 30,000 patients [28].

Hence, one of the explanations for the differences in outcomes between the two ethnic groups is the contribution of LDL levels. Despite the fact that Arabs had higher mean LDL levels, they were treated less aggressively with statin therapy. This is supported by the findings of our study showing that higher LDL levels were independently associated with a greater risk of CV mortality (HR 1.031, *p* = 0.037). The contribution of high LDL levels to the disparities in outcomes is further reinforced because, although the recommendations for treatment was similar in both ethnic groups, Arab recipients demonstrated a lower use of statins than Jews (68% vs. 92% for Arabs and Jews, respectively; *p* < 0.001).

Differences in healthy life-styles are also evident between the two populations and contribute to higher CV morbidity and mortality. Physical activity in accordance with the WHO recommendations has been reported to be significantly lower in the Arab population (43.7% vs. 28.7% of Jewish and Arabs men respectively; 31.9% vs. 18.1% of Jewish and Arab women respectively) as reported in the National Health Survey [30]. Hence, controlling risk factors with an emphasis on LDL levels and statin therapy is of utmost importance [31, 32].

All multivariate models were extended to include wider combinations of different variables (Supplemental Table), ethnicity remained significantly associated with outcomes, with some reduction in the ethnicity coefficient. It is possible that they are simply confounders, yet, as not all possible confounders were recorded or adjusted for, the contribution of the above-mentioned risk factors (i.e., healthy lifestyles, physical activity, nutritional elements) cannot be eliminated.

Another explanation for the differences in outcomes between the two ethnic groups, could be related to the fact that Arabs are more likely than Jews to receive a heart from an ethnic mismatched donor and hence perhaps less genetically match.

### Health policies implications

The data presented in this paper will help to design treatment protocols and regimens unique to our patients, emphasizing on frequency of biopsies, immunosuppressive protocols, medication adherence assessment, and subsequent close monitoring.

Further identifying the causes of Arabs excess cardiovascular risk may help provide enhanced surveillance and culturally sensitive care. Future, prospectively designed trials, are required in order to define additional factors that may explain these differences (focusing on social, ethnic, medication adherence and nutritional variables). This should be prioritized by public health systems and by organ transplant programs. These data call for the establishment of a National Heart Transplant Registry.

Increased awareness and early intervention by the Israeli healthcare system, such as the responsible national authorities and public health providers, and cooperation with the Arab community is of paramount importance. Efforts should target dedicated and individualized education of patients; families; primary care medical staff; religious leaders such as Imams and heads of families, with consideration of the unique family fabric in Arab society. Implementation of prevention programs to reduce cardiovascular risk factors, particularly diabetes and obesity, may help reduce the disparity between Arabs and Jews. Individualizing the cardiovascular follow up protocol emphasizing on early vasculopathy assessment including frequent oriented visits and more intense use of non-invasive cardiovascular screening modalities (i.e., donor-specific antibodies screening, ergometry, stress echocardiography, myocardial perfusion imaging) for early detection of cardiac vasculopathy.

### Study limitations

The major limitation of our study lies in its observational nature. The relevance of the various social determinants of health (i.e., socioeconomic status, health awareness) and modifiable risk factors for CAV and CV mortality (i.e., BMI, physical activity, dietary details) were not assessed in this study and therefore deserve further investigation. Compliance was only indirectly evaluated based on patient adherence with recommended follow up protocols. However, pill count and other formal methods for adherence assessment were not available. Our current practice does not include routine intravascular ultrasound (IVUS) assessment which might be associated with underestimation of CAV. While the sample size might be too small to draw definitive conclusions, our study provides the most current data and the largest report on ethnic disparities between Arabs and Jews undergoing HTx in Israel.

## Conclusions

Our data from a tertiary Israeli HTx Registry show important baseline clinical differences between Arabs and Jews in Israel who undergo HTx and suggest that Arab ethnicity is associated with a pronounced and significant increase in the risk for CAV and CV mortality following HTx. Further studies are required to evaluate whether more aggressive risk factor management in the Israeli Arab population following HTx would reduce CAV and CV mortality in this high-risk population. Increased awareness and early intervention of the Israeli healthcare system and cooperation with the Arab community is of paramount importance, and will undoubtedly lead to improved outcomes after HTx.

## Abbreviations

ARS: Any rejection score
CAV: Cardiac allograft vasculopathy
CV: Cardiovascular
EMB: Endomyocardial biopsy
HTx: Heart transplantation
ISHLT: International Society of Heart and Lung Transplantation
TRS: Total rejection score
